# Pharmacotherapy with Fluoxetine Restores Functional Connectivity from the Dentate Gyrus to Field CA3 in the Ts65Dn Mouse Model of Down Syndrome

**DOI:** 10.1371/journal.pone.0061689

**Published:** 2013-04-19

**Authors:** Fiorenza Stagni, Jacopo Magistretti, Sandra Guidi, Elisabetta Ciani, Chiara Mangano, Laura Calzà, Renata Bartesaghi

**Affiliations:** 1 Department of Biomedical and Neuromotor Sciences, University of Bologna, Bologna, Italy; 2 Department of Biology and Biotechnology “L. Spallanzani”, University of Pavia, Pavia, Italy; 3 Health Sciences and Technologies - Interdepartmental Center for Industrial Research (HST-ICIR), University of Bologna, Bologna, Italy; IGBMC/ICS, France

## Abstract

Down syndrome (DS) is a high-incidence genetic pathology characterized by severe impairment of cognitive functions, including declarative memory. Impairment of hippocampus-dependent long-term memory in DS appears to be related to anatomo-functional alterations of the hippocampal trisynaptic circuit formed by the dentate gyrus (DG) granule cells - CA3 pyramidal neurons - CA1 pyramidal neurons. No therapies exist to improve cognitive disability in individuals with DS. In previous studies we demonstrated that pharmacotherapy with fluoxetine restores neurogenesis, granule cell number and dendritic morphology in the DG of the Ts65Dn mouse model of DS. The goal of the current study was to establish whether treatment rescues the impairment of synaptic connectivity between the DG and CA3 that characterizes the trisomic condition. Euploid and Ts65Dn mice were treated with fluoxetine during the first two postnatal weeks and examined 45–60 days after treatment cessation. Untreated Ts65Dn mice had a hypotrophyc mossy fiber bundle, fewer synaptic contacts, fewer glutamatergic contacts, and fewer dendritic spines in the stratum lucidum of CA3, the terminal field of the granule cell projections. Electrophysiological recordings from CA3 pyramidal neurons showed that in Ts65Dn mice the frequency of both mEPSCs and mIPSCs was reduced, indicating an overall impairment of excitatory and inhibitory inputs to CA3 pyramidal neurons. In treated Ts65Dn mice all these aberrant features were fully normalized, indicating that fluoxetine can rescue functional connectivity between the DG and CA3. The positive effects of fluoxetine on the DG-CA3 system suggest that early treatment with this drug could be a suitable therapy, possibly usable in humans, to restore the physiology of the hippocampal networks and, hence, memory functions.

## Introduction

Down syndrome (DS) is a high-incidence genetic pathology caused by triplication of human chromosome 21. Individuals with DS may have various medical problems, but intellectual disability is the unavoidable characteristic and the most invalidating aspect of this pathology. Mental retardation has been related to the decreased brain size of DS individuals, a feature that is already apparent early in development. Accumulating evidence in DS mouse models clearly shows severe neurogenesis impairment in the major brain neurogenic regions (see [1]), suggesting that defective neurogenesis may be a key determinant of brain hypotrophy and mental retardation. Similarly to the Ts65Dn mouse model of DS, human fetuses with DS exhibit proliferation impairment in various brain regions [2]–[4], which validates the use of this model to study trisomy-linked brain alterations. Evidence in humans and mouse models of DS shows severe dendritic alterations that appear to correlate with the cognitive profile [1], [5], [6]. Though defective neurogenesis is probably a crucial determinant of mental retardation, dendritic hypotrophy and spine density reduction with consequent connectivity alterations are also likely to be important actors.

An impairment of declarative memory, that starts from childhood and is retained in adulthood, stands out as one of the cognitive defects associated with DS [7]–[9]. Mouse models of DS exhibit a similar impairment of hippocampus-dependent memory functions [10]–[13]. The granule cells of the dentate gyrus (DG), pyramidal neurons of field CA3 and pyramidal neurons of field CA1 form the major circuit of the hippocampal formation, the so-called trisynaptic circuit. Neocortical signals from polymodal cortices are relayed to the DG by the entorhinal cortex. The processing of neocortical signals along the trisynaptic circuit is essential for long-term declarative memory. Histological studies have shown various structural abnormalities in the hippocampal formation of individuals with DS and in mouse models of DS. The DG and hippocampus of fetuses with DS have fewer neurons than normal fetuses [3]. Likewise, the DG of Ts65Dn mice has fewer granule cells across all postnatal life stages [2], [12], [14], [15]. In contrast, the number of hippocampal pyramidal cells is not reduced in adulthood [14] and in aged Ts65Dn mice the CA3 field has more neurons compared to that of controls [15]. Spine density is reduced in granule cells of the DG [6], [16] and CA3 pyramidal neurons [17] and synapse-to-neuron ratio are reduced in the DG and hippocampus of adult Ts65Dn mice [18]. Recordings from the DG have shown no alterations in the basic properties of evoked synaptic responses in Ts65Dn mice, though long-term potentiation (LTP) is impaired due to an increase in the overall GABAergic synaptic input impinging on the granule cells [19]. In field CA3 of Ts65Dn mice the frequency of miniature EPSCs is reduced, indicating an overall impoverishment of afferent synaptic input from the DG [20], which is in agreement with the reduced spine density [17] and the reduced number of granule neurons [1] in trisomic mice.

The anatomo-functional alterations mentioned above suggest that altered signal processing by the trisynaptic circuit underlies memory impairment in DS. Very few studies have explored the possibility of pharmacologically improving neurodevelomental defects in DS during early developmental stages. Based on evidence that the serotonergic system, which plays a key role in neurogenesis and dendritic development, is altered in the trisomic brain, we previously attempted a therapy with fluoxetine, a selective serotonin reuptake inhibitor [12]. We found that in neonate Ts65Dn mice treated with fluoxetine there was a full recovery of neurogenesis of granule cell precursors in the DG, consistently with evidence from adult Ts65Dn mice [21], and total number of granule cells. These effects were accompanied by a full recovery of hippocampus-dependent memory performance [12]. The reduced granule cell number in trisomic mice implies a reduction in the amount of excitatory signals transferred to field CA3. Field CA3 is thought to play a major role in both pattern completion and separation during memory storage [22]. The restoration of granule cell number by treatment with fluoxetine suggests a possible normalization of the input from the dentate gyrus to CA3 which may take part in the restoration of memory performance found in trisomic mice treated with fluoxetine [12]. Since there is no evidence that the altered functional connections between DG and CA3 in the trisomic brain can be pharmacologically restored, the goal of the current study was to investigate the effects of early treatment with fluoxetine on the functional synaptic connectivity between the DG and CA3 in Ts65Dn mice.

## Materials and Methods

### Colony

Female Ts65Dn mice carrying a partial trisomy of chromosome 16 [23] were obtained from Jackson Laboratories (Bar Harbour, ME, USA) and maintained on the original genetic background by mating them with C57BL/6JEi x C3SnHeSnJ (B6EiC3) F1 males. Animals were karyotyped using real-time quantitative PCR (qPCR) as previously described [24]. Genotyping was validated with fluorescent *in situ* hybridization (FISH) [25]. The day of birth was designed as postnatal day (P) zero. A total of 90 mice were used. The animals' health and comfort were controlled by the veterinary service. The animals had access to water and food *ad libitum* and lived in a room with a 12:12 hour dark/light cycle. Experiments were performed in accordance with the Italian and European Community law for the use of experimental animals and were approved by Bologna University Bioethical Committee (Permit Number: 16154-X/10). In this study all efforts were made to minimize animal suffering and to keep the number of animals used to a minimum.

### Experimental protocol

**Figure 1:**
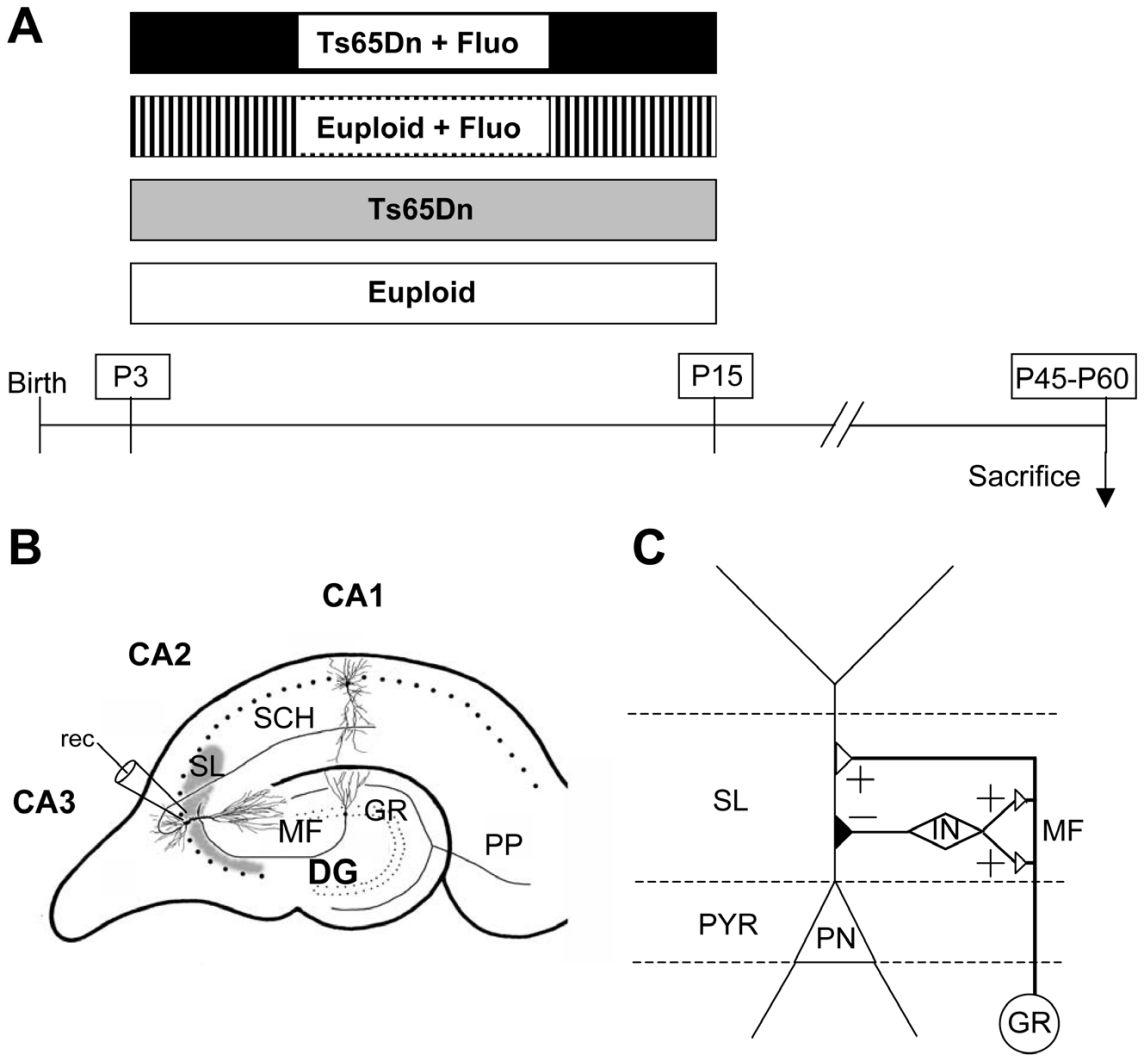
Experimental protocol. A: Euploid and Ts65Dn mice received a daily injection of either saline (Eu; Ts65Dn) or fluoxetine (Eu+Fluo; Ts65Dn+Fluo) from postnatal (P) day 3 to P15. Animals were killed on P45–P60 and used for evaluation of connectivity (P45) between the dentate gyrus and field CA3 and electrophysiological recordings from CA3 (P45–P60). B: Schematic drawing of a section across the hippocampal formation showing the major intrinsic connections. Patch clamp recording (rec) of miniature synaptic potentials were carried out from pyramidal neurons of field CA3. The area occupied by the mossy fiber terminals in the stratum lucidum of field CA3 is indicated in gray. C: Mossy fiber circuitry in CA3. Mossy fibers establish excitatory synapses (+) with pyramidal neurons and inhibitory interneurons in the stratum lucidum. Inhibitory interneurons establish inhibitory synapses (−) with the pyramidal neurons. Abbreviations: CA1-3, hippocampal fields; DG, dentate gyrus; IN, inhibitory interneuron; MF, mossy fivers; PN, pyramidal neuron; PP, perforant pathway; PYR, pyramidal layer; SCH, Shaffer collaterals; SL, stratum lucidum.

Euploid (*n* = 23) and Ts65Dn (*n* = 19) mice received a daily subcutaneous injection (at 9–10am) of fluoxetine (Sigma-Aldrich) in 0.9% NaCl solution from P3 to P15 (dose: 5 mg/kg from P3 to P7; 10 mg/kg from P8 to P15). We chose a maximum daily dose of 10 mg/kg because, due to the extremely short half-life of fluoxetine in rodents compared to humans, such a dose is thought to produce a brain concentration of a magnitude similar to that of 20–60 mg taken daily by humans [26], [27]. Age-matched euploid (*n* = 29) and Ts65Dn (*n* = 20) mice were injected with the vehicle (Fig. 1A). Each treatment group had approximately the same composition of males and females. At the age of 45 days animals were divided into groups and used as indicated below. The animals from the first group (*n* = 4–8 for each experimental condition) were used for electrophysiological recordings from field CA3. Those in the second group (n = 4–7 for each experimental condition) were transcardially perfused with ice-cold phosphate-buffered saline (PBS) followed by a 4% solution of paraformaldehyde in PBS. Brains were stored in the fixative for 24 h, cut along the midline and placed in a 20% sucrose in phosphate buffer solution for an additional 24 h. Hemispheres were frozen and stored at −80°C. The left and right hemispheres were cut with a freezing microtome in 30-µm-thick coronal sections that were serially collected in antifreeze solution containing sodium azide. Sections from the right hemisphere were used for polysialylated neural cell adhesion molecule (PSA-NCAM) immunohistochemistry and sections from the left hemisphere were used for synaptophysin (SYN) and vesicular glutamate transporter 1 (VGLUT1) immunohistochemistry. Animals from the third group (n = 5–9 for each experimental condition) were not perfused; brains were quickly removed, cut along the midline, rinsed in PBS and Golgi stained. These animals were the same as those used in a previous study [16]. Animals belonging to the fourth group (n = 5 for each experimental condition) were not perfused; brains were quickly removed, the hippocampal region was dissected and kept at −80°C; one hippocampus from each animal was used for RT-qPCR analysis and the other was used for Western blotting.

### Patch-clamp experiments: preparation of slices

Mice were anaesthetised by inhalation of isoflurane (Merial Italia, Milan, Italy) and decapitated. The brain was quickly extracted under hypothermic conditions and submerged in an ice-cold artificial cerebro-spinal fluid (ACSF) composed of (in mmol·l^−1^): 125 NaCl, 3 KCl, 24 NaHCO_3_, 1.25 KH_2_PO_4_, 1.2 MgSO_4_, 2 CaCl_2_, 10 d-glucose (pH 7.4 by saturation with 95% O_2_, 5% CO_2_). Two coronal cuts were made, in order to remove the anterior half and the occipital pole of the brain, and the piece thus obtained was laid on the posterior section plane. The tissue was blocked on the stage of a Microslicer DTK-1000 vibratome (Dosaka, Kyoto, Japan) using cyanoacrilate glue. During the sectioning procedure the tissue was submerged in an ice-cold (∼1°C) cutting solution containing (in mmol·l^−1^): 130 K-gluconate, 15 KCl, 20 N-2-hydroxyethyl piperazine-N-2-ethanesulphonic acid (HEPES), 0.2 EGTA, 11 d-glucose (pH 7.4 with KOH). The use of this high-K^+^ solution was found to improve neuron viability (Stéphane Dieudonné, unpublished results). 350-µm thick sagittal sections across the dorsal hippocampus were cut, then rinsed in ACSF and transferred to an incubation chamber filled with the same solution (continuously bubbled with 95% O_2_, 5% CO_2_). The slices were kept submerged in the incubation chamber at room temperature for at least one hour before the recording was started.

### Patch-clamp experiments: voltage-clamp recordings, drugs, and data analysis

Whole-cell, patch-clamp recordings from CA3 pyramidal neurons were carried out on acute slices of the dorsal hippocampus obtained as described above. The experimental set-up employed and the basic procedures followed were the same as described elsewhere [28]. Briefly, cells were visualized by means of an upright microscope (Axioskop 2 FS; Zeiss, Oberkochen, FRG) equipped with a ×60 water-immersion objective lens, differential-contrast optics, and a near-infrared charge-coupled device (CCD) camera. Slices were perfused with ACSF (continuously bubbled with 95% O_2_, 5% CO_2_) at a rate of about 1.5 ml/min. Patch pipettes were fabricated from thick-wall borosilicate glass capillaries (CEI GC 150–7.5; Harvard Apparatus, Edenbridge, UK) by means of a Sutter P-87 horizontal puller (Sutter Instruments, Novato, CA, USA). The pipette solution contained (in mmol/l): 150 CsF, 4 CsCl, 2 MgCl_2_, 10 N-2-hydroxyethyl piperazine-N-2-ethanesulphonic acid (HEPES), 10 ethylene glycol-bis (β-aminoethyl ether) N,N,N′,N′-tetraacetic acid (EGTA), 2 adenosine 5′-triphosphate (ATP)-Na_2_, 0.2 guanosine 5′-triphosphate (GTP)-Na, 2.5 lidocaine N-ethyl bromide (QX-314) (pH adjusted to 7.2 with CsOH). The patch pipettes had a resistance of 3–5 MΩ when filled with the above solution. Tight seals (>5 GΩ) and the whole-cell configuration were obtained by suction according to the standard technique (see Castelli *et al.*, 2007). Voltage-clamp recordings of Na^+^ currents were performed at room temperature (21–22°C) by means of an Axopatch 200B patch-clamp amplifier (Axon Instruments, Foster City, CA, USA). Series resistance (*R*_s_) was evaluated on line by canceling the whole-cell capacitive transients evoked by −5-mV voltage square pulses with the amplifier built-in compensation section, and reading out the corresponding values. *R*_s_ was normally 5–12 MΩ and always <20 MΩ, and was compensated by ∼90%. Current signals were acquired in gap-free modality with a personal computer interfaced to a Digidata 1322A interface (Axon Instr.) using the Clampex program of the pClamp 8.2 software package (Axon Instr.). Current signals were low-pass filtered at 5 kHz and digitized at 20 kHz.

All drugs applied were preliminarily dissolved in concentrated aliquots and stored at −20°C, then re-dissolved to the final concentrations in ACSF and delivered to the recorded cells via the general perfusion. Tetrodotoxin (TTx) was purchased from Alomone Labs. (Jerusalem, Israel), 1(S),9(R)-(−)-bicuculline methiodide and QX-314 were purchased from Sigma-Aldrich S.r.l. (Milan, Italy), and 2,3-dioxo-6-nitro-1,2,3,4-tetrahydrobenzo[*f*]quinoxaline-7-sulfonamide (NBQX), D-(-)-2-amino-5-phosphonopentanoic acid (APV), and (2*S*,2′*R*,3′*R*)-2-(2′,3′-dicarboxycyclopropyl)glycine (DCG-IV) from Tocris (Bristol, UK).

mEPSCs and mIPSCs recorded under the conditions specified in the Results were off-line detected using an automated threshold routine in the LabView environment written and kindly provided by Dr. G. Biella (University of Pavia). All detected events were also visually inspected one-by-one for confirmation or rejection.

### Histological procedures

#### Golgi staining

Brains were Golgi-stained using the FD Rapid Golgi Stain ™ Kit (FD NeuroTechnologies, Inc.). Brains were immersed in the impregnation solution containing mercuric chloride, potassium dichromate and potassium chromate and stored at room temperature in darkness for 3 weeks. Hemispheres were cut with a microtome in 90-µm-thick coronal sections that were mounted on gelatin-coated slides and were air-dried at room temperature in the dark for one day. After drying, sections were rinsed with distilled water and subsequently stained in a developing solution (FD Rapid Golgi Stain Kit).

#### SYN and VGLUT1 immunohistochemistry

Free-floating sections (n = 3–4 per animal) from the hippocampal formation were submitted to double fluorescence immunohistochemistry for SYN and VGLUT1 immunohistochemistry. For SYN and VGLUT1, immunohistochemistry sections were incubated for 48 h at 4°C with mouse monoclonal anti-SYN (SY38) antibody (Millipore Bioscience Research Reagents) and rabbit polyclonal anti-VGLUT1 (Abcam) antibody both diluted 1∶1000. Sections were then incubated overnight at 4°C with a DyLight-conjugated goat anti-mouse IgG (Thermo Scientific) and a RRX-conjugated donkey anti-rabbit IgG (Jackson Laboratory) both diluted 1∶100.

#### *PSA-NCAM immunohistochemistry*

Free-floating sections (n = 3–4 per animal) were permeabilized with 0.1% Triton X-100 in PBS, blocked for 1 hour in 1.5% Goat Serum in 0.1% Triton X-100 and PBS, incubated overnight at 4°C with an anti-Polysialic Acid-NCAM mouse monoclonal antibody (Chemicon), diluted 1∶100; incubated for 2 h with a peroxidase-conjugated AffiniPure goat anti-Mouse IgM secondary antibody (Jackson ImmunoResearch), diluted 1∶200; reacted with diaminobenzidine and rinsed in water, mounted on glass slides and dehydrated with a gradient of alcohols, cleared and cover slipped.

### Real-time reverse transcriptase quantitative PCR (RT-qPCR)

Total RNA was extracted from the hippocampus of euploid and Ts65Dn mice with TriReagent (Sigma) according to the manufacturer's instructions. cDNA synthesis was achieved with 1.0 µg of total RNA using the iScript™ cDNA Synthesis Kit (Bio-Rad) according to the manufacturer's instructions. The primer sequences used are the following: (i) brain-derived neurotrophic factor (BDNF) (NM_007540.4), forward 5′- GTGACAGTATTAGCGAGTG-3′ and reverse 5′- GCCTTCCTTCGTGTAACC-3′; (ii) glyceraldehyde-3-phosphate dehydrogenase (GAPDH) (NM_008084.2), forward 5′-GAACATCATCCCTGCATCCA-3′ and reverse 5′-CCAGTGAGCTTCCCGTTCA-3′. Real-time PCR was performed using a SYBR *Premix Ex Taq kit* (Takara, Shiga, Japan) according to the manufacturer's instructions in an iQ5 real time PCR detection system (Bio-Rad). Fluorescence was determined at the last step of every cycle. Real-time PCR assay was done under the following universal conditions: 2 min at 50°C, 10 min at 95°C, 50 cycles of denaturation at 95°C for 15 sec, and annealing/extension at 60°C for 1 min. Relative quantification was performed using the ΔΔCt method.

### Western blotting

Total proteins from the hippocampal formation were obtained as previously described [29] and protein concentration was estimated using the Lowry method. Proteins (20 µg) were subjected to electrophoresis on a 4–20% Mini-PROTEAN® TGX™ Precast Gel (BioRad) and transferred to a Hybond ECL nitrocellulose membrane (Amersham Life Science). The following primary antibodies were used: anti-SYN (1∶1000; Millipore Bioscience Research Reagents); anti-VGLUT1 (1∶1000; ABCAM) and anti- GAPDH (1∶5000; Sigma). Densitometric analysis of digitized images was performed with Scion Image software (Scion Corporation, Frederick, MD, USA), and intensity for each band was normalized to the intensity of the corresponding GAPDH band.

### Measurements

#### Neuron sampling

Golgi-stained neurons were sampled across the rostro-caudal extent of field CA3 (n = 10–15 sections per animal). The total number of sampled neurons was 6–8 per animal. Only well-impregnated neurons were chosen for the histological analysis. Sampling was made on coded slides so that the drawer was not aware of the animal's treatment.

#### Spine density

The axons of the granule cells project to field CA3 and make synaptic contacts with the thorny excrescences located on the proximal part of the dendritic shaft of CA3 pyramidal neurons. The thorny excrescences are protrusions on the dendritic shaft and are formed by 2–5 spine-like structures. Spines of CA3 pyramidal neurons were counted using a 100× oil immersion objective lens. The length of the shaft covered by spinous excrescences was determined and the number of individual spines of the thorny excrescences was counted manually. The linear spine density was calculated by dividing the total number of spines by the length of the dendritic segment. Spine density was expressed as number of spines per 20 µm dendrite.

#### Thickness of the mossy fiber bundle

The axons of the granule cells, the mossy fibers, can be visualized with PSA-NCAM immunohistochemistry [30]–[32]. In each sampled section, the area of the region occupied by the mossy fibers in field CA3 was first measured by tracing its contour. The length of this area was then obtained by tracing a line at its border with the pyramidal layer of field CA3. The thickness of the mossy fiber system increases, progressing from the hilus of the dentate gyrus toward field CA2. To estimate the mean thickness of the system over field CA3, we divided the area occupied by mossy fibers by its length. Measurements of individual sections were averaged in each animal to obtain the mean thickness of the mossy fiber terminal field.

#### Connectivity in the stratum lucidum of field CA3

To study the innervation of the apical dendrites of CA3 pyramidal neurons in the stratum lucidum, the terminal field of the mossy fibers, intensity of SYN immunoreactivity (IR) or VGUT1 was determined by optical densitometry of immunohistochemically stained sections (n = 3–4 per animal). Fluorescence images were captured using a Nikon Eclipse E600 microscope equipped with a Nikon Digital Camera DXM1200 (ATI system). Densitometric analysis of SYN or VGLUT1 was carried out using Nis-Elements Software 3.21.03 (Nikon). A box of 490 µm^2^ was used and placed in the stratum lucidum of CA3. Six measurements were taken in each section. For each image, the intensity threshold was estimated by analyzing the distribution of pixel intensities in the image areas that did not contain IR. This value was then subtracted to calculate IR of each sampled area.

#### Glutamatergic innervation of the stratum lucidum of field CA3

Dual-channel confocal microscopy was used to study co-localization of SYN with VGLUT1. Sections were scanned with a Nikon Ti-E fluorescence microscope coupled with an A1R confocal system (Nikon). The lasers were: Multi-Ar (457/488/514) with exciting wavelengths for DyLight 488 and 561 diode-pumped solid state laser (DPSS) with exciting wavelengths for RRX. The conditions for co-localization analysis were the following: the objective was an oil immersion ×60 objective (n.a. 1.4); laser power was kept low in order to avoid photobleaching; the zoom factor was 6; software Nis-Elements AR 3.2 was used and image size was 512×512 pixels. In each section three images from the stratum lucidum were taken. For each image, the intensity thresholds were estimated by analyzing the distribution of pixel intensities in the image areas that did not contain IR. This value, the background threshold, was then subtracted, and the green-red co-localization coefficient was calculated.

#### *Density of synaptophysin immunoreactive puncta in the stratum lucidum of field CA3*

From the same sections indicated above, we evaluated the density of individual puncta exhibiting SYN immunoreactivity in the stratum lucidum (image size; 512×512 pixels; three images per section).

### Statistical analysis

Results are presented as the mean ± SD of the mean. Data from single animals were the unity of analysis. Statistical testing was performed with ANOVA followed by *post hoc* comparisons with the Duncan or Bonferroni test. A probability level (*p*) lower than 0.05 was considered to be statistically significant.

## Results

### Effect of fluoxetine on the size of the mossy fiber bundle in euploid and Ts65Dn mice

**Figure 2:**
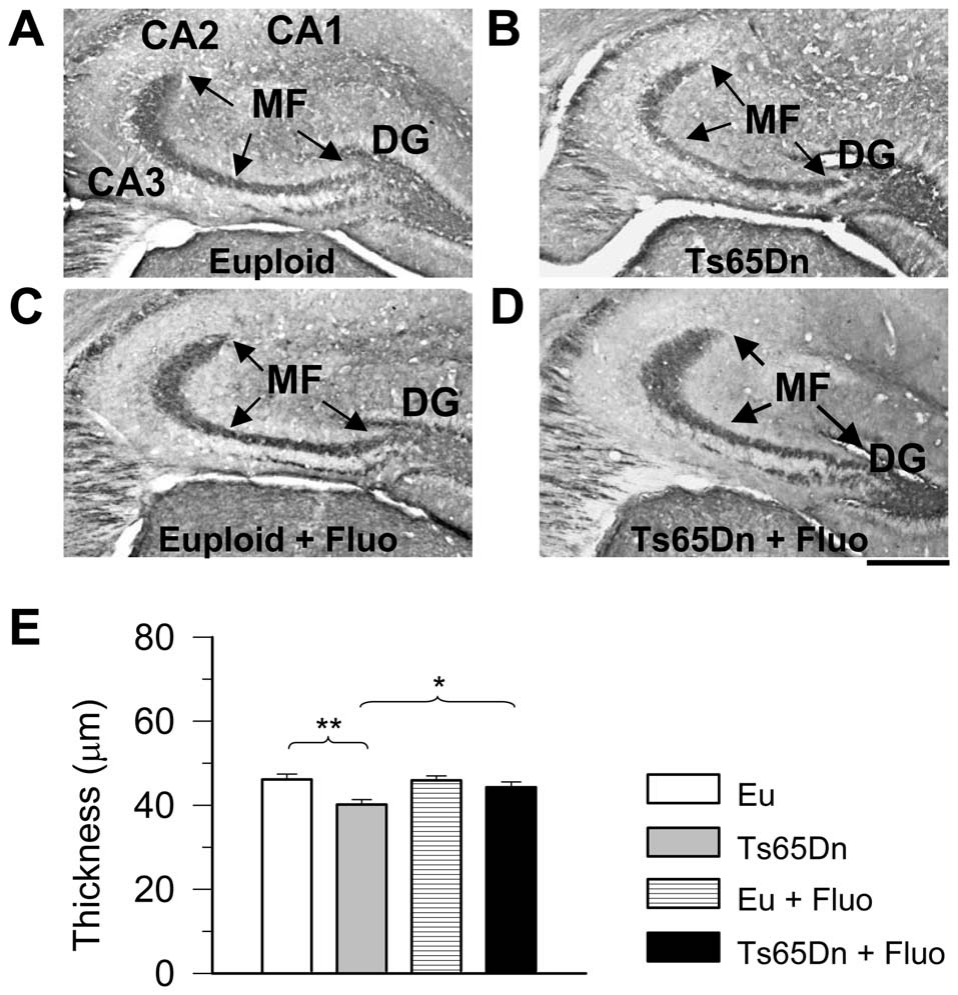
Effect of fluoxetine on the thickness of the mossy fiber terminal field. A–D: Examples of coronal sections, processed for PSA-NCAM immunohistochemistry, across the hippocampal formation of an animal from each of the following experimental groups: euploid (A), Ts65Dn (B), treated euploid (C), and treated Ts65Dn (D). Sections were taken at approximately the same distance from the rostral border of the hippocampal formation. The mossy fiber system (indicated by arrows) can be clearly recognized for its dark color and its abrupt termination at the border between CA3 and CA2. The scale bar = 300 µm applies to A–D. Abbreviations: DG, dentate gyrus; MF, mossy fibers. C: Mean thickness of the mossy fiber terminal field in CA3 in untreated euploid (n = 7), untreated Ts65Dn (n = 6), treated euploid (n = 6) and treated Ts65Dn (n = 4) mice. The mean thickness was obtained, from 3–4 sections per animal, by dividing the area occupied by the mossy fibers by the length of CA3. Values represent mean ± SD. * p<0.05; ** p<0.01 (Duncan's test after ANOVA).

The granule cells send their axons, the mossy fibers, to field CA3. The region of synaptic contact forms a layer called the stratum lucidum (Fig. 1B). The mossy fiber axons express PSA-NCAM [30]–[32], which makes it possible to examine this bundle in sections immunoprocessed for PSA-NCAM. Ts65Dn mice have a reduced number of granule cells in comparison with euploid mice [12], which implies a reduction in the number of mossy fibers reaching the stratum lucidum. A reduced number of fibers may result in a reduced thickness of the terminal field of the mossy fibers in field CA3. To obtain information regarding this issue we examined the size of the mossy fiber system in the stratum lucidum of field CA3. Fig. 2A,B shows that the mossy fiber system was patently less prominent in Ts65Dn than in euploid mice. Quantification of the mean thickness of the mossy fiber bundle within CA3 showed that in Ts65Dn mice it was smaller (−13%) than in euploid mice (Fig. 2E). In euploid mice, treatment with fluoxetine did not change the mean thickness of the mossy fiber bundle (Fig. 2A–E). Importantly, in trisomic mice treated with fluoxetine the thickness of the mossy fiber bundle increased and became similar to that of untreated euploid mice (Fig. 2A–E).

### Effect of fluoxetine on overall innervation in the stratum lucidum of euploid and Ts65Dn mice

**Figure 3:**
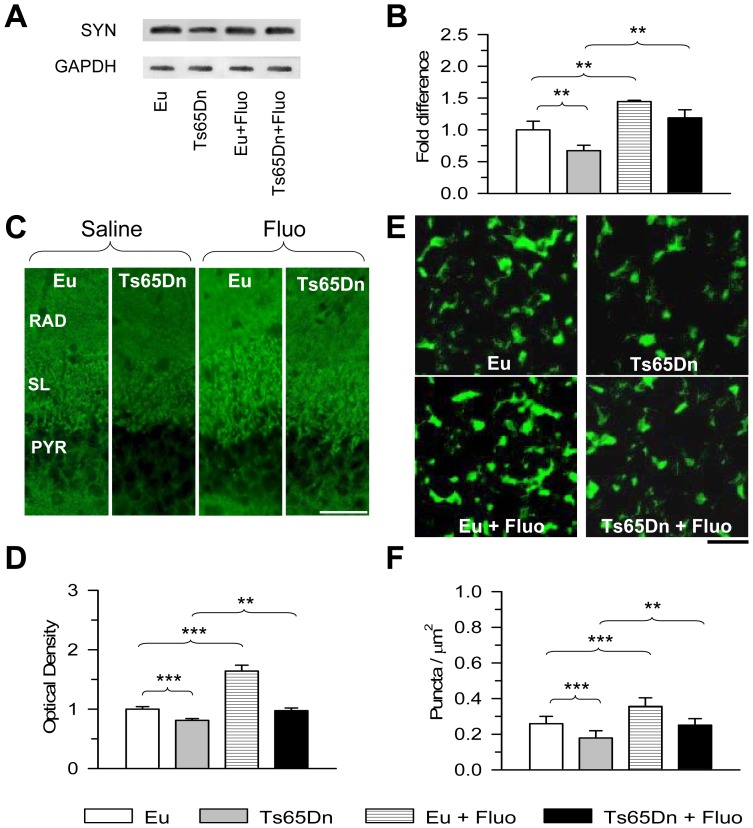
Effect of fluoxetine on overall innervation in stratum lucidum of field CA3. A–B: Western blot analysis of synaptophysin levels in the hippocampal formation of untreated euploid (n = 5), untreated Ts65Dn (n = 5), treated euploid (n = 5) and treated Ts65Dn (n = 5) mice. Western immunoblots in (A) are examples from an animal of each experimental group. Histograms in (B) show synaptophysin levels normalized to GAPDH and expressed as fold difference in comparison with untreated euploid mice. C: Images of sections processed for synaptophysin immunofluorescence from field CA3 from an animal of each experimental group. Scale bar = 50 µm. D: Optical density of synaptophysin immunoreactivity in the stratum lucidum of untreated euploid (n = 7), untreated Ts65Dn (n = 6), treated euploid (n = 6) and treated Ts65Dn (n = 4) mice. Number of analyzed sections: 3–4 per animal. Six measurements were taken from each section in a box of 490 µm^2^, randomly placed in the stratum lucidum of CA3. Data are given as fold difference vs. the stratum lucidum of untreated euploid mice. E: Images, taken with the confocal microscope, of sections processed for synaptophysin immunofluorescence from the stratum lucidum of field CA3 from an animal of each experimental group. Scale bar = 5 µm. F: Number of puncta per µm^2^ exhibiting synaptophysin immunoreactivity in untreated euploid (n = 7), untreated Ts65Dn (n = 6), treated euploid (n = 6) and treated Ts65Dn (n = 4) mice. The density of individual puncta exhibiting synaptophysin immunoreactivity was evaluated in 3–4 sections per animal (image size; 512×512 pixels; three images per section). Values in B, D and F represent mean ± SD. ** p<0.01; *** p<0.001 (Duncan's test after ANOVA). Abbreviations: Eu, euploid; Fluo, fluoxetine; PYR, stratum pyramidale; RAD, stratum radiatum; SL, stratum lucidum.

Synaptophysin (SYN; also known as p38) is an integral membrane glycoprotein of synaptic vesicles that is a specific marker of presynaptic terminals. Evaluation of SYN levels in the hippocampal formation (dentate gyrus plus hippocampus proper) using Western blot analysis showed that Ts65Dn mice had reduced SYN levels that were restored with treatment (Fig. 3A,B). SYN levels also increased in treated euploid mice and became higher than in untreated euploid mice (Fig. 3A,B). Next, in order to obtain information on overall connectivity to the stratum lucidum, we examined the immunoreactivity for SYN in this layer. Fig. 3C shows representative images from animals of each group. It can be readily appreciated that the immunoreactivity for SYN was reduced in Ts65Dn mice compared to untreated euploid mice and that treatment with fluoxetine increased SYN immunoreactivity both in Ts65Dn and euploid mice. Quantitative analysis showed that in untreated Ts65Dn mice the optical density (OD) of SYN was significantly lower (−20%) than in untreated euploid mice. In Ts65Dn mice treated with fluoxetine, the OD of SYN underwent a major increase and became similar to that found in untreated euploid mice (Fig. 3D). An increase in the OD of SYN also took place in treated euploid mice with an increase of 60% in comparison with untreated euploid mice (Fig. 3D).

Differences in SYN immunoreactivity among groups may be attributable to differences in the number of synaptic terminals and/or to differences in the number of synaptic vesicles contained in each terminal. To establish possible differences among groups in the overall number of synaptic contacts, we evaluated the density of individual puncta exhibiting SYN immunoreactivity in the stratum lucidum. Fig. 3E shows representative images from animals of each experimental group. We found that untreated Ts65Dn mice had fewer puncta (−31%) exhibiting SYN immunoreactivity than the euploid counterparts (Fig. 3F), suggesting that fewer synapses overall are established on CA3 pyramidal neurons. Treatment with fluoxetine increased the density of SYN puncta both in Ts65Dn and euploid mice (Fig. 3F). A comparison between treated Ts65Dn mice and untreated euploid mice showed no differences between these groups, suggesting that fluoxetine had induced a recovery in the number of synaptic terminals. A comparison between treated and untreated euploid mice showed that the former had significantly more SYN puncta (+38%) than the latter (Fig. 3F), suggesting an increase in the number of synaptic terminals also in euploid mice.

### Effect of fluoxetine on glutamatergic innervation in the stratum lucidum of euploid and Ts65Dn mice

**Figure 4:**
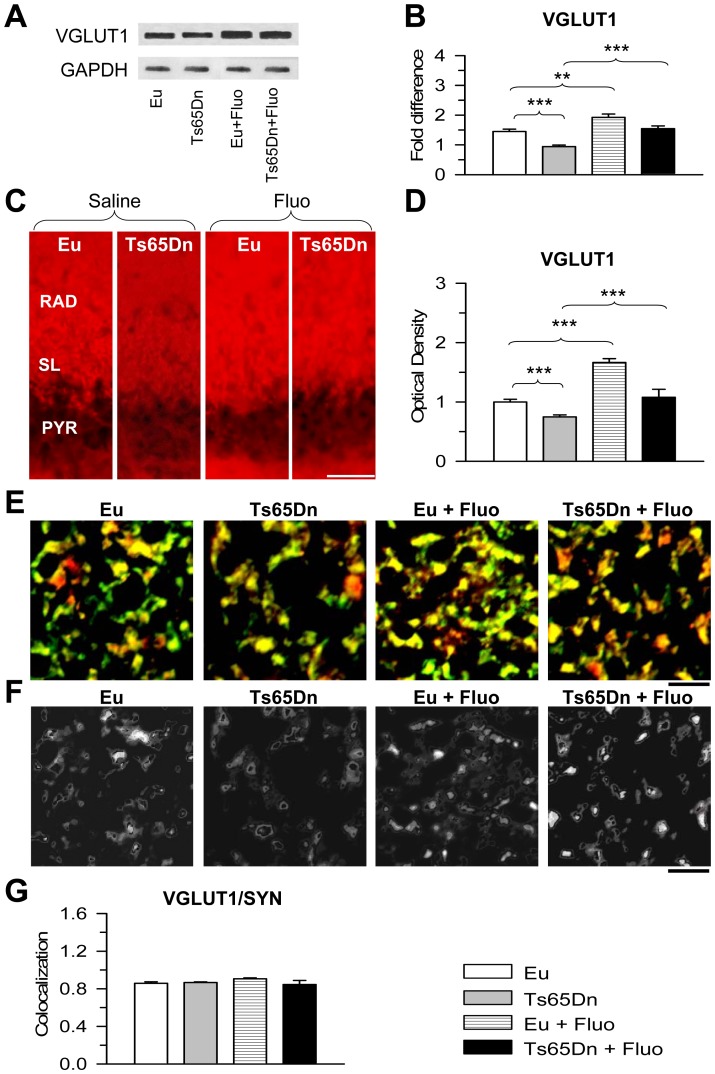
Effect of fluoxetine on the glutamatergic innervation in stratum lucidum of field CA3. A–B: Western blot analysis of VGLUT1 levels in the hippocampal formation of untreated euploid (n = 5), untreated Ts65Dn (n = 5), treated euploid (n = 5) and treated Ts65Dn (n = 5) mice. Western immunoblots in (A) are examples from an animal of each experimental group. Histograms in (B) show VGLUT1 levels normalized to GAPDH and expressed as fold difference in comparison with untreated euploid mice. C: Images of sections processed for VGLUT1 immunofluorescence from field CA3 of an animal of each experimental group. Scale bar = 50 µm. D: Optical density of VGLUT1 immunoreactivity in the stratum lucidum of untreated euploid (n = 7), untreated Ts65Dn (n = 6), treated euploid (n = 6) and treated Ts65Dn (n = 4) mice. Number of analyzed sections: 3–4 per animal. Six measurements were taken from each section in a box of 490 µm^2^, randomly placed in the stratum lucidum of CA3. Data are given as fold difference vs. stratum lucidum of untreated euploid mice. E, F: Images, taken with the confocal microscope, of sections processed for double-labeling immunofluorescence with an anti-synaptophysin antibody (green) and an anti-VGLUT1 antibody (red) from the stratum lucidum of an animal from each experimental group. Images in (F) represent colocalization (white) between these two markers. Images correspond to the same images shown in Figure 3E. Scale bar = 5 µm. G: Coefficient of colocalization of synaptophysin (SYN) and VGLUT1 in stratum lucidum of untreated euploid (n = 7), untreated Ts65Dn (n = 6), treated euploid (n = 6) and treated Ts65Dn (n = 4) mice. The coefficient of colocalization was evaluated in 3–4 sections per animal (image size; 512×512 pixels; three images per section). Values in D and G represent mean ± SD. *** p<0.001 (Duncan's test after ANOVA). Abbreviations: Eu, euploid; Fluo, fluoxetine; PYR, stratum pyramidale; RAD, stratum radiatum; SYN, synaptophysin; SL, stratum lucidum.

The granule cells use glutamate as a neurotransmitter. Their axons form large complex “mossy” synapses on the thorny excrescences covering the proximal apical dendritic shaft of CA3 pyramidal neurons. In addition, the granule cell axons give origin to thin philopodial extensions and small en passant boutons onto local circuit inhibitory interneurons primarily located within the stratum lucidum [33], [34] (Fig. 1C). To establish the effect of trisomy and treatment on the glutamatergic input we evaluated the glutamate vesicular transporter 1 (VGLUT1) levels in the hippocampal formation (dentate gyrus plus hippocampus proper) using Western blot analysis. Results showed that Ts65Dn mice had reduced VGLUT1 levels that were restored with treatment (Fig. 4A,B). VGLUT1 levels also increased in treated euploid mice and became higher than in untreated euploid mice (Fig. 4A,B). To specifically establish the effect of trisomy and treatment on the glutamatergic terminals from the DG, we evaluated immunoreactivity for the glutamate vesicular transporter 1 (VGLUT1) in the stratum lucidum of treated and untreated mice. Fig. 4C shows representative images from animals of each group. It can be noted that the immunoreactivity for VGLUT1 was reduced in Ts65Dn mice compared to untreated euploid mice and that treatment with fluoxetine increased VGLUT1 immunoreactivity both in Ts65Dn and euploid mice. In untreated Ts65Dn mice the OD of VGLUT1 was significantly lower (−25%) than in untreated euploid mice (Fig. 4D). In Ts65Dn mice treated with fluoxetine, the OD of VGLUT1 underwent an increase and became similar to that found in untreated euploid mice (Fig. 4D). The OD of VGLUT1 also increased by 66% in treated euploid mice compared with untreated euploid mice (Fig. 4D).

The stratum lucidum harbors, in addition to excitatory projections from the granule cells of the DG, inhibitory interneurons that impinge upon the proximal shaft of field CA3 pyramidal neurons [33], [34]. In order to establish the effect of trisomy and treatment on the relative abundance of glutamatergic terminals we examined the co-localization of SYN and VGLUT1 in the stratum lucidum of treated and untreated mice. Fig. 4E shows representative images processed for double-labeling immunofluorescence with an anti-synaptophysin antibody (green) and an anti-VGLUT1 antibody (red) and Fig. 4F shows the co-localization (white) of these markers. We found no differences among groups in the co-localization of SYN and VGLUT1 (Fig. 4G). The co-localization coefficient had a high value (approximately 0.8), which is consistent with the prevalence of glutamateric projections to the stratum lucidum. The similarity in the co-localization coefficient between euploid and Ts65Dn mice indicates that, though in trisomic mice there were fewer synaptic terminals, the ratio of glutamatergic (excitatory) and non-glutamatergic (inhibitory) synapses was similar to that of euploid mice. Likewise, in trisomic and euploid mice treated with fluoxetine, the finding that the co-localization coefficient was unchanged in comparison with the untreated counterparts indicates that the overall increase in the number of synaptic contacts involved, in a proportional manner, excitatory and inhibitory synapses.

### Effect of fluoxetine on spine density on the proximal shaft of CA3 pyramidal neurons in euploid and Ts65Dn mice

**Figure 5:**
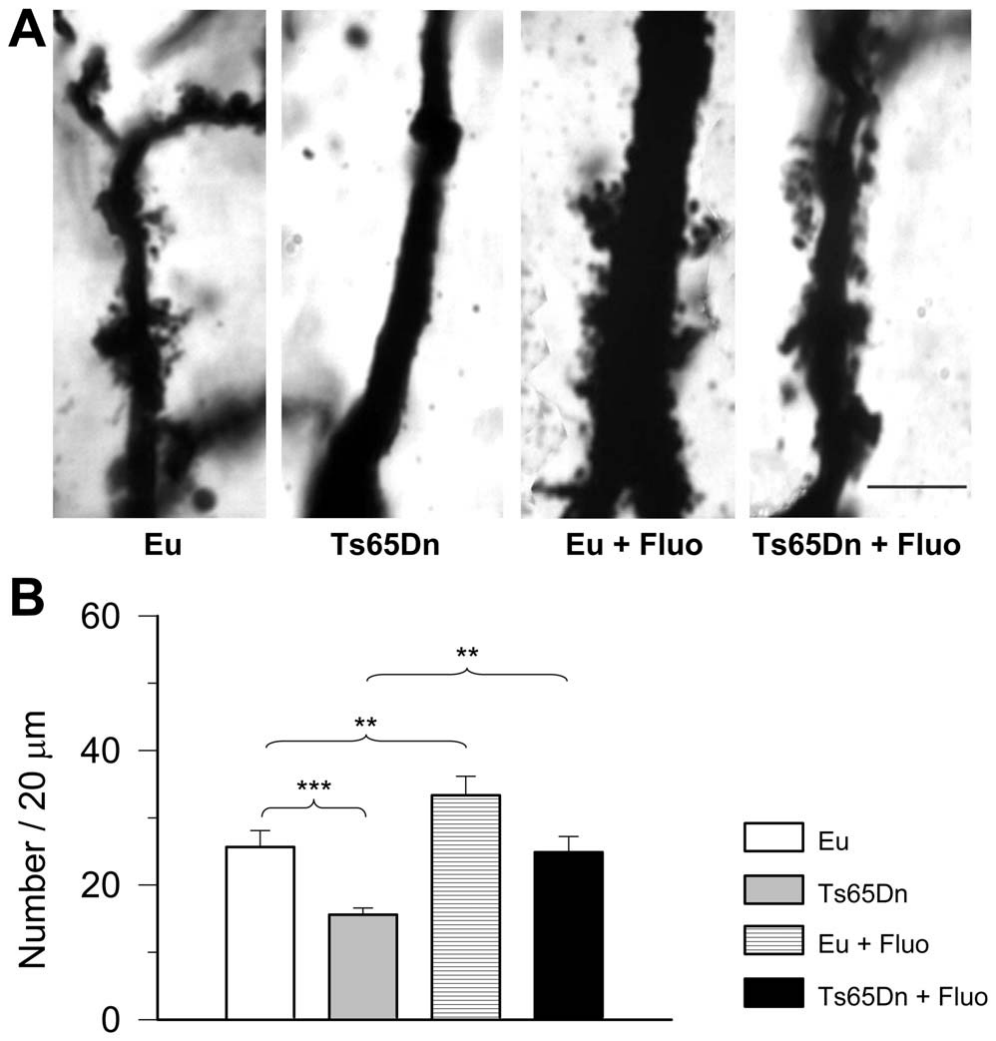
Effect of fluoxetine on CA3 pyramidal neuron spine density. A: Photomicrograph of Golgi-stained field CA3 pyramidal cells showing the spinous excrescences on the proximal apical dendritic shaft in an animal from each experimental group. Scale bar = 10 µm. B: Density of spines on the proximal apical dendritic shaft in the stratum lucidum of field CA3 of untreated euploid (n = 9), untreated Ts65Dn (n = 5), treated euploid (n = 5) and treated Ts65Dn (n = 5) mice. Individual spines of the thorny excrescences were counted over the length of the dendritic shaft covered by spinous excrescences in 6–8 neurons per animal. Spine density is expressed as number of spines per 20 µm of dendrite. Values represent mean ± SD. * p<0.05; ** p<0.01; *** p<0.001 (Duncan's test after ANOVA).

The mossy fiber axons establish multiple asymmetrical synapses with the thorny excrescences on the proximal portion of the apical dendritic tree of CA3 pyramidal neurons. Recent evidence shows a decreased number of thorns on the thorny excrescences of Ts65Dn mice aged six months [17]. We were interested in establishing whether a reduction in the density of spines forming the thorny excrescences is already present in mice aged 45 days and whether fluoxetine rescues this defect. Images in Fig. 5A show that in untreated Ts65Dn mice there were patently fewer spines compared to euploid mice and that treatment increased their number. An increase in spine density also took place in treated euploid mice (Fig. 5A). Quantification of spine density showed that in untreated Ts65Dn mice spine density was notably lower (−40%) than that of untreated euploid mice (Fig. 5B). After treatment with fluoxetine, Ts65Dn mice underwent a notable increase in spine density that became similar to that of untreated euploid mice (Fig. 5B). Treatment also induced a significant increase in spine density in euploid mice (+30% compared to untreated mice).

### Effect of fluoxetine on basal synaptic input in CA3 neurons

“Miniature” synaptic events reflect the spontaneous release of neurotransmitters from all presynaptic terminals converging on the recorded neuron. The frequency of these events is related to the total number of presynaptic terminals and the probability of release at each terminal. To functionally evaluate the basal excitatory and inhibitory synaptic input to CA3 pyramidal neurons, we recorded spontaneous miniature excitatory postsynaptic currents (mEPSCs) and miniature inhibitory postsynaptic currents (mIPSCs) from individual pyramidal neurons by performing whole-cell, patch-clamp experiments in the voltage-clamp mode. Miniature events were recorded in the presence of tetrodotoxin (TTx, 1 µM) in the perfusing solution, so as to prevent spontaneous synaptic events due to presynaptic action-potential firing. In each cell, mEPSC and mIPSC activity was continuously recorded for a period of at least 5 min and up to 10 min.

**Figure 6:**
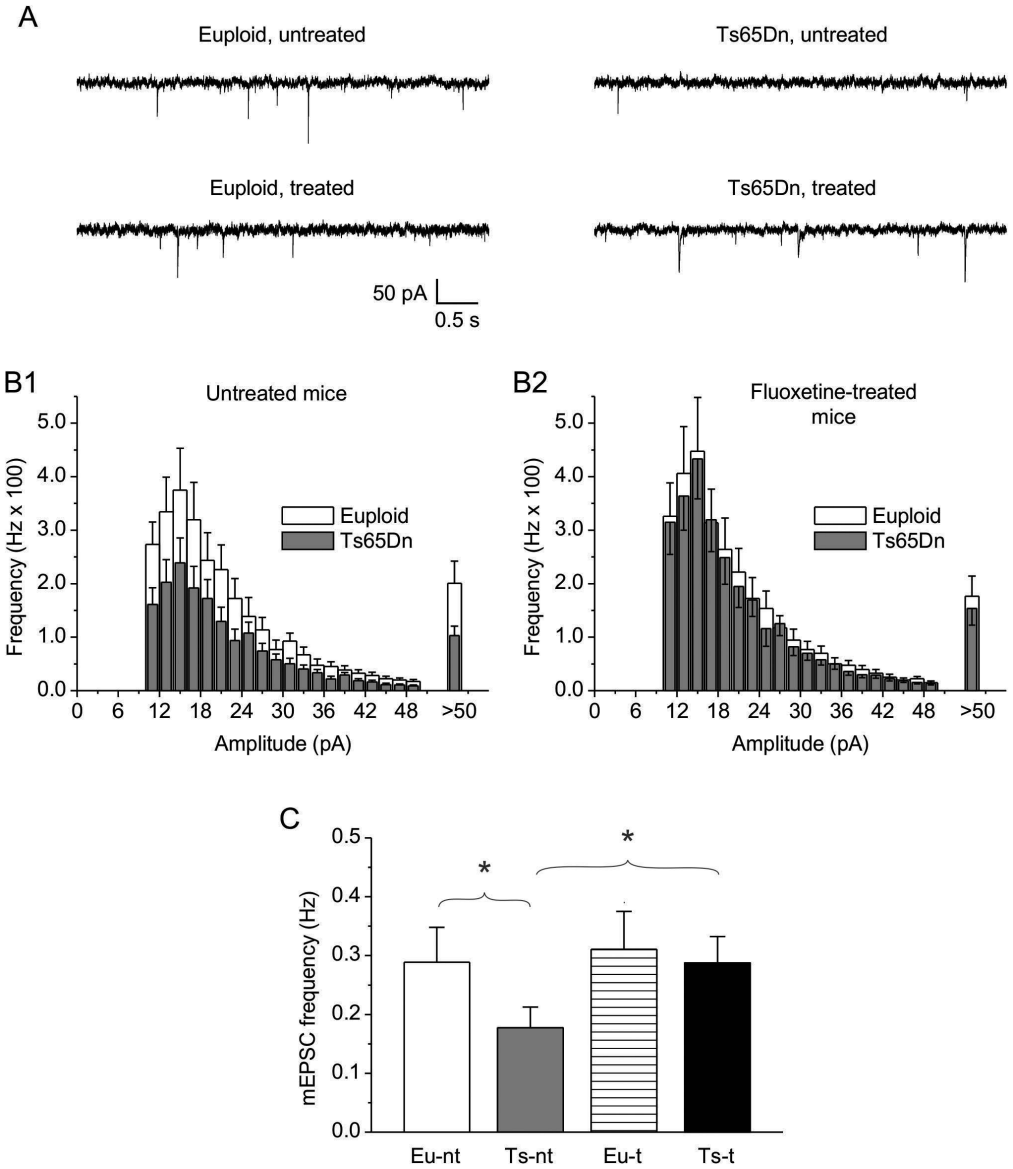
Effects of fluoxetine on mEPSC frequency in CA3 pyramidal neurons. A: Exemplary current tracings recorded in the gap-free mode in four representative cells from untreated euploid and Ts65Dn mice and euploid and Ts65Dn mice treated with fluoxetine, showing mEPSC activity. Holding potential was −70 mV. Recordings were made in the presence of 1-µM TTx in the superfusing solution. B: Average frequency-distribution diagrams of mEPSC amplitude for untreated euploid and Ts65Dn mice (B1) and euploid and Ts65Dn mice treated with fluoxetine (B2). Numbers of observations are: 8 (euploid, untreated mice), 4 (Ts65Dn, untreated mice), 7 (euploid, fluoxetine-treated mice), and 5 (Ts65Dn, fluoxetine-treated mice) (these values indicate the number of animals of each group from which recordings were obtained; 2 to 4 cells were recorded from each animal). C: Average, overall mEPSC frequency in the four animal groups (*n*: as above). One-way ANOVA revealed the existence of a statistically significant difference among groups (*p* = 0.007). *, *p*<0.05 (Bonferroni post-test; the same test also revealed a significant difference, at the 0.01 level, between Ts65Dn, untreated mice and euploid, fluoxetine-treated mice).

For mEPSC recording, the holding potential was set at −70 mV, a level that, under our experimental conditions, was close to the theoretical equilibrium potential of chloride ions (−71.5 mV), and therefore to the reversal potential of GABAergic currents: this allowed for mEPSC recording in virtual isolation from mIPSCs. Fig. 6A shows examples of mEPSCs recorded, under the above conditions, in representative cells from untreated and fluoxetine-treated euploid and trisomic mice. No spontaneous synaptic events were observable any longer after application of the glutamatergic inhibitors, NBQX (10 µM)+APV (50 µM) (*n* = 4 cells; not shown). Due to baseline noise levels normally observed at −70 mV, events of less than 10 pA in peak amplitude were ignored. Accepted events were then used to construct frequency-distribution diagrams of mEPSC amplitude. Data were averaged among the cells from each animal (*n* = 2 to 4), and then among the animals pertaining to the same experimental group (untreated euploid or trisomic mice, fluoxetine-treated euploid or trisomic mice). The plots thus obtained are shown in Fig. 6B. In untreated mice, the trisomic condition was associated with a clear reduction in mEPSC frequency for all amplitude classes, with no evident changes in frequency-distribution shape (Fig. 6B1), in accordance with previous observations [20]. The overall mEPSC frequency was significantly reduced in trisomic animals by ∼39% (Fig. 6C). The frequency distribution of mEPSC amplitude was also qualitatively similar in fluoxetine-treated mice (Fig. 6B2). The overall mEPSC frequency was found to be slightly, albeit non-significantly, higher in fluoxetine-treated euploid mice than in untreated euploid mice. However, in fluoxetine-treated trisomic mice it was significantly higher than in untreated trisomic mice and not significantly different from the mEPSC frequency found in euploid untreated or treated mice (Fig. 6C).

**Figure 7:**
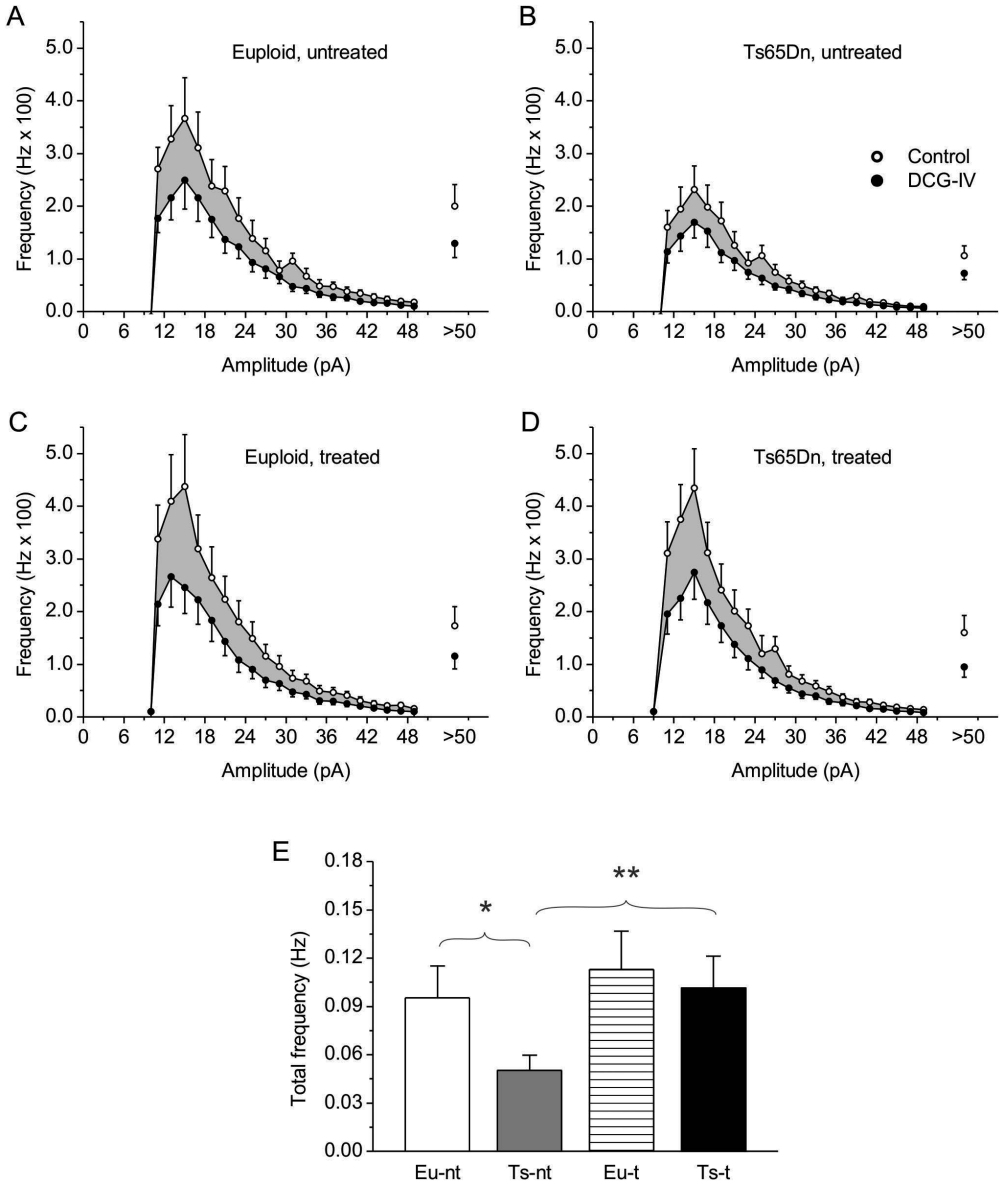
Effects of fluoxetine on mEPSCs due to mossy-fiber input to CA3 pyramidal neurons. A–D: Average frequency-distribution diagrams of mEPSC amplitude for untreated euploid (A) and Ts65Dn (B) mice, and fluoxetine-treated euploid (C) and Ts65Dn (D) mice, before (open symbols) and during (filled symbols) application of the group-II metabotropic glutamate receptor (mGluR) agonist, DCG-IV (3 µM) plus 50-µM APV. The shadowed areas correspond to the frequency of the mEPSC activity removed by this drug treatment. Numbers of observations are: 7 (euploid, untreated mice), 4 (Ts65Dn, untreated mice), 6 (euploid, fluoxetine-treated mice), and 5 (Ts65Dn, fluoxetine-treated mice) (these values have the same meaning as explained in Fig. 8 legend). E: Average, overall frequency of the mEPSC activity removed by DCG-IV+APV treatment, obtained by subtraction, in the four animal groups (*n*: as above). One-way ANOVA revealed the existence of a statistically significant difference among groups (*p*<0.001). * and **, *p*<0.05 and 0.01, respectively (Bonferroni post-test; the same test also revealed a significant difference, at the 0.001 level, between Ts65Dn, untreated mice and euploid, fluoxetine-treated mice).

The above data show that in trisomic animals fluoxetine treatment increased the basal, excitatory synaptic input to pyramidal CA3 neurons to nearly normal levels. The sources of this synaptic input include both granule-cell mossy fibers and association afferents from other pyramidal neurons. To ascertain whether the synaptic input specifically due to mossy fibers was restored in fluoxetine-treated trisomic animals, we adopted the same experimental strategy described by Hanson *et al.* [20]. In a subset of cells of each animal group, after mEPSC recording under basal conditions, the group-II metabotropic glutamate receptor (mGluR) agonist, DCG-IV (3 µM) was applied in association with the NMDA-receptor antagonist, APV (50 µM). Under these conditions, group-II mGluR activation is known to selectively inhibit synaptic transmission in mossy-fiber inputs [20] but not in associational connections [35]. DGC-IV+APV application markedly reduced mEPSC frequency at all amplitude classes in each animal group (Fig. 7A). The mEPSC activity abolished by DGC-IV, quantified by subtracting the overall mEPSC frequency in the presence of DCG-IV+APV from the overall mEPSC frequency in control conditions, was significantly reduced in untreated trisomic animals as compared to euploid animals, but was restored to nearly normal levels in fluoxetine-treated trisomic animals (Fig. 7B). These results suggest that fluoxetine treatment was able to recover functionally normal synaptic inputs from mossy fibers in CA3 pyramidal neurons.

**Figure 8:**
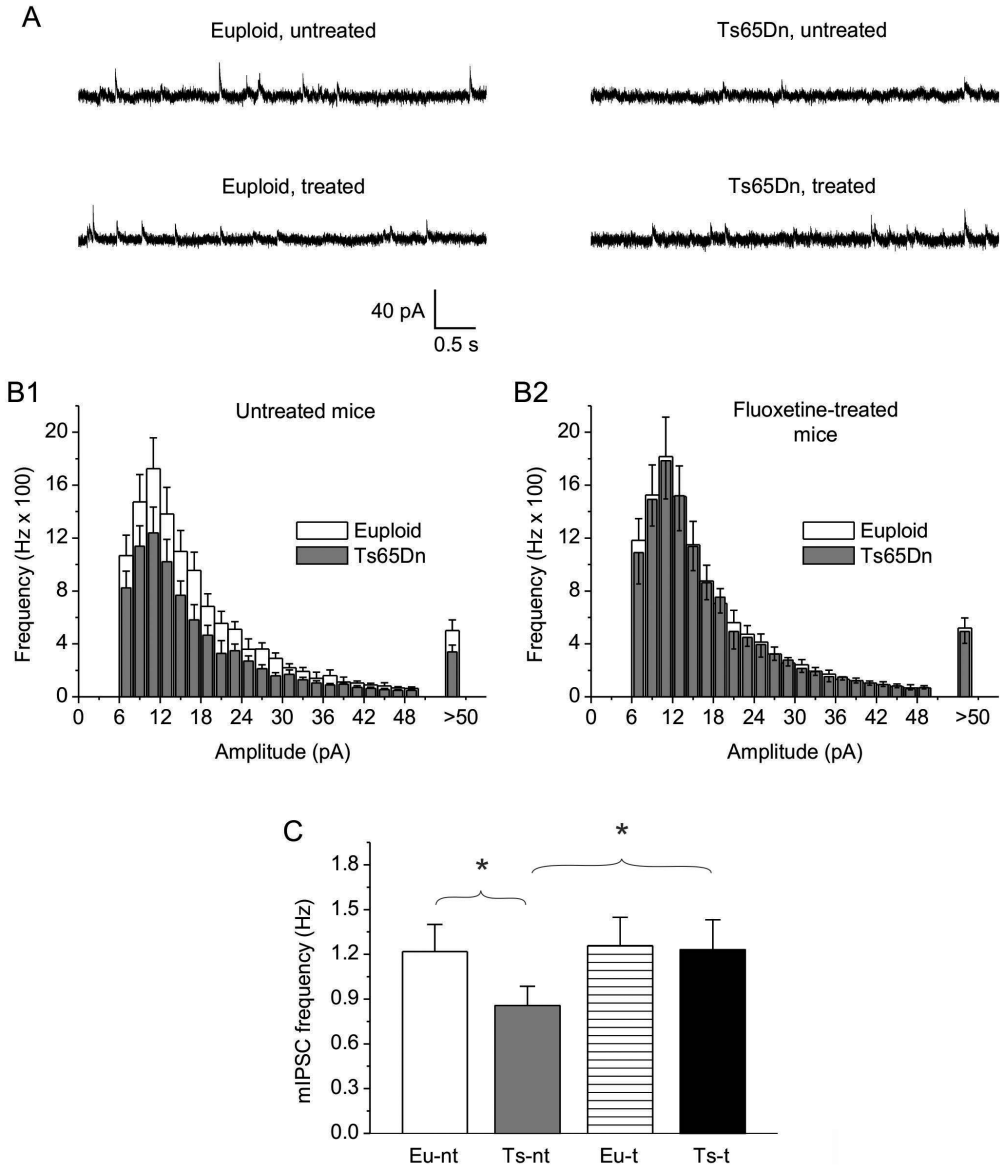
Effects of fluoxetine on mIPSC frequency in CA3 pyramidal neurons.

mIPSCs were recorded by setting the holding potential at 0 mV, a level close to the reversal potential of glutamatergic currents, which allowed for virtual elimination of visible mEPSC activity. Examples of mIPSCs recorded in representative cells from the four animal groups considered are shown in Fig. 8A. mIPSC activity was abolished by application of the GABA_A_-ergic inhibitor, bicuculline (20 µM) (*n* = 3 cells; not shown). Detected events of >6 pA in peak amplitude were used to construct frequency-distribution diagrams of mIPSC amplitude, and data were averaged among cells from each animal (*n* = 2 to 4) and then among animals, as above (Fig. 8B). In untreated mice, the trisomic condition was associated with a significant reduction (∼−30%) in mIPSC frequency (Fig. 8B1, C), again in accordance with previous observations [20]. Fluoxetine treatment of trisomic mice restored mIPSC frequency to levels not significantly different from those observed in euploid animals (Fig. 8B2, C).

### Effect of fluoxetine on BDNF expression

**Figure 9:**
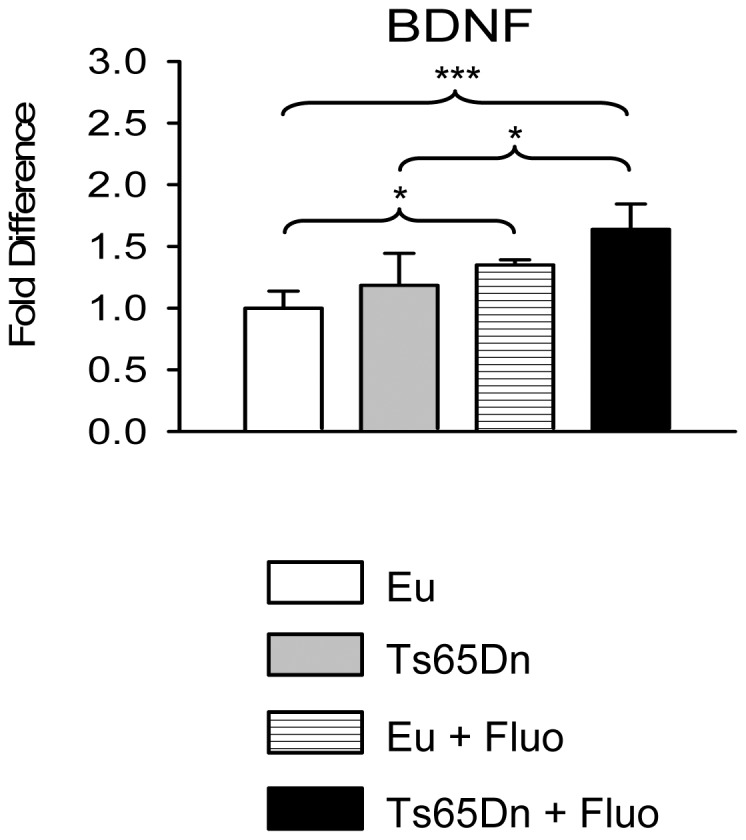
Effect of fluoxetine on hippocampal BDNF levels. RNA expression levels of BDNF, quantified by RT-qPCR, in homogenates of the hippocampal formation from untreated euploid (n = 5), untreated Ts65Dn (n = 5), treated euploid (n = 5) and treated Ts65Dn (n = 4) mice. Data (mean ± SD) are given as fold difference in comparison with untreated euploid mice. p<0.05; *** p<0.001 (Duncan's test after ANOVA).

Neurotrophins and, in particular, the brain-derived neurotrophic factor (BDNF) are important regulators of neuronal plasticity. Synaptic reorganization mediated by BDNF is thought to be a critical process which shapes neuronal networks. Reduced BDNF expression has been documented in the frontal cortex of Ts65Dn mice [36]. We previously found that in the hippocampus of Ts65Dn mice aged 15 days BDNF expression was lower than in euploid mice and that BDNF levels were restored by treatment with fluoxetine [12]. This suggests that reduced BDNF levels may take part in developmental alterations in the trisomic brain and that restoration of BDNF levels may be a determinant of the beneficial effects of treatment with fluoxetine. We evaluated here BDNF levels in the hippocampus of mice aged 45 days. At this age, we found no differences in BDNF levels between untreated euploid and Ts65Dn mice (Fig. 9). Normal BDNF levels have been detected in the hippocampus of adult mice, [37] suggesting that failure of BDNF production is not a permanent defect in the trisomic hippocampus. However, after treatment with fluoxetine in the period P3-P15, Ts65Dn mice and euploid mice aged 45 days exhibited higher BDNF levels than the untreated counterparts (Fig. 9). This increase was approximately +35% in treated vs. untreated euploid mice, and +38% in treated vs. untreated Ts65Dn mice. The increase in BDNF levels in treated mice is consistent with the effects of antidepressants on brain BDNF expression [38].

## Discussion

This study shows that in Ts65Dn mice the connectivity from the granule cells to field CA3 is impaired and that treatment with fluoxetine restores the anatomy and function of the mossy fiber system, in parallel with total granule cell number. In view of the key role of CA3 in declarative memory, this effect is an essential event for the recovery of the trisynaptic network properties and, hence, of the hippocampus-dependent memory performance.

### Impaired connectivity in the stratum lucidum of field CA3 of Ts65Dn mice

In Ts65Dn mice, consistently with the reduced number of granule cells in the DG [12] and, hence, a reduction in mossy fiber axons, the thickness of the mossy fiber bundle in the stratum lucidum of CA3 was reduced in comparison with that of euploid mice. In agreement with previous evidence [18], we found that Ts65Dn mice had overall fewer synaptic terminals and fewer glutamatergic terminals in the stratum lucidum. The density reduction of the glutamatergic terminals is consistent with the reduced number of granule cells that are the source of the glutamatergic input to the stratum lucidum. Since excitatory inputs terminate on dendritic spines, the reduced spine density on the thorny excrescences of trisomic mice is the counterpart of the reduced density of the glutamatergic terminals. Consistently with the hypotrophy of the glutamatergic system in the stratum lucidum, in Ts65Dn mice mEPSC frequency was notably reduced. Taken together these data indicate a reduced excitatory drive from the granule cells to the pyramidal neurons of CA3 and, consequently, impaired signal processing. The mossy fibers additionally innervate inhibitory interneurons in the stratum lucidum that exert a feedforward inhibitory control on pyramidal neuron discharge [33], [34]. If the reduction in the density of the glutamatergic terminals in the stratum lucidum of Ts65Dn mice includes synapses on inhibitory interneurons, this will result in a reduction in feedforward inhibition, matching the reduction in direct excitation. In field CA3 of Ts65Dn mice there was a reduction in the frequency of mIPSCs, which is in line with the reduction in the density of non-glutamatergic synapses in the stratum lucidum and suggests a reduced inhibitory control on CA3 pyramidal neurons. It is important to note that, though in absolute terms Ts65Dn mice had fewer excitatory and inhibitory synaptic contacts, the ratio of these inputs was similar to that of euploid mice. This confirms that in field CA3, unlike in the dentate gyrus [19], a shift towards over-inhibition is not present [20].

### Fluoxetine restores the mossy fiber projections and functional connectivity in the stratum lucidum of Ts65Dn mice

In agreement with the treatment-induced restoration of total granule cell number [12], the thickness of the mossy fiber bundle of treated Ts65Dn mice underwent an increase and became similar to that of euploid mice. Since synaptogenesis and neuron maturation take place in the first few postnatal weeks, treatment with fluoxetine during this critical time window is expected to have a significant impact on altered connectivity in the trisomic brain. Consistently with this idea, we found that, after treatment with fluoxetine during the first two postnatal weeks, Ts65Dn mice showed an increase in the overall number of synaptic terminals in the stratum lucidum. This increase was due to a proportionally similar increase in the number of glutamatergic and non-glutamatergic terminals. In parallel, in Ts65Dn mice there was an increase in spine density on the proximal shaft of CA3 pyramidal neurons in the stratum lucidum. These two effects were accompanied by an increase in the frequency of mEPSC, indicating that the new connections were functionally effective. Importantly, in treated Ts65Dn mice there was also an increase in the frequency of mIPSCs, indicating an increase in the inhibitory input to the pyramidal cells. In the stratum lucidum of field CA3, feedforward inhibition is fundamental for shaping the pattern and duration of pyramidal neuron discharge in response to the mossy fiber input [34]. A reduction in feedforward inhibition would lead to excessive recruitment of pyramidal cells and predisposition to epileptiform activity. It appears, thus, of relevance that in treated trisomic mice the increase in the number of excitatory synapses was matched by a parallel increase in the inhibitory input.

### Fluoxetine only marginally increases functional connectivity in the stratum lucidum of euploid mice

The thickness of the mossy fiber bundle was not affected by treatment in euploid mice, which fits with the absence of a significant increase in total granule cell number [12]. In treated euploid mice, the large increase in synaptophysin immunoreactivity in the stratum lucidum was accompanied by a more moderate increase in the number of synaptophysin immunoreactive puncta and the large increase in VGLUT1 immunoreactivity was accompanied by a less prominent increase in spine density. Taken together this evidence suggests that in euploid mice fluoxetine, in addition to increasing the number of synaptic contacts on CA3 pyramidal neurons, may increase the number of synaptic vesicles in individual synapses. The similarity in the colocalization coefficient of synaptophysin and VGLUT1 between treated and untreated euploid mice suggests that, similarly to trisomic mice, treatment increased glutamatergic and non-glutamatergic terminals in a proportional manner. In treated euploid mice, the frequency of mEPSCs and mIPSCs underwent a moderate but not statistically significant increase, suggesting that among the surplus of synaptic terminals there were silent terminals. The overall scarce effect of treatment on functional connectivity in field CA3 of euploid mice is in line with the moderate effects of fluoxetine on granule neurons of the DG and the lack of an effect on hippocampus-dependent memory performance [12], [16].

### BDNF may contribute to synaptic remodeling in field CA3 of Ts65Dn and euploid mice treated with fluoxetine

During the formation of new mossy fiber synapses, mossy fibers contact the dendritic shaft of CA3 pyramidal neurons and induce postsynaptic modifications that culminate in the formation of complex giant spines [39]. This suggests that the increase in the number of mossy fibers in treated Ts65Dn mice may favor the increase in spine density (and synaptic boutons) in the stratum lucidum. Though in euploid mice fluoxetine did not substantially increase the number of granule neurons/mossy fibers [12], it induced an increase in the density of glutamatergic terminals and dendritic spines. This implies that “non-anatomical” mechanisms may be involved in the generation of additional synapses in the stratum lucidum of euploid mice. BDNF is one of the most potent modulators of synaptic plasticity and spine formation [40], [41]. The mossy fiber pathway contains the highest levels of BDNF in the CNS, and BDNF appears to regulate both direct excitatory and indirect inhibitory inputs to CA3 pyramidal cells [42]. The increase in BDNF levels in mice treated with fluoxetine suggests that BDNF may underlie the sprouting of glutamatergic terminals in the stratum lucidum of euploid mice and may concur, in conjunction with the increase in the number of mossy fibers, to favor the formation of glutamatergic synapses in Ts65Dn mice. BDNF is also present in mossy fiber terminals contacting inhibitory neurons [42], suggesting that the increase in the inhibitory innervation in the stratum lucidum of treated euploid and Ts65Dn mice may also be mediated by mossy fiber BDNF.

### Conclusions

**Figure 10:**
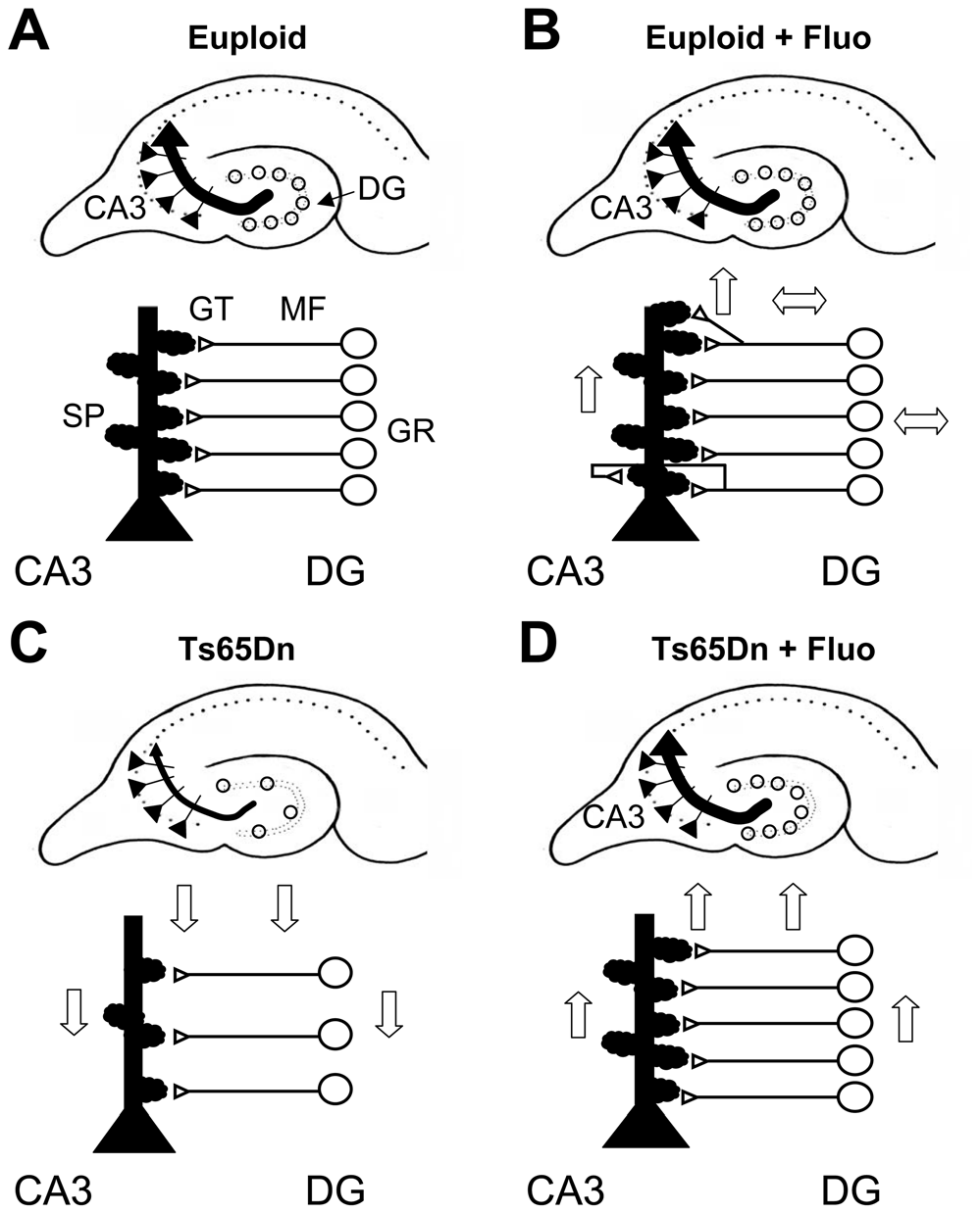
Summary of the effect of fluoxetine on connectivity in the stratum lucidum. A–D: Connectivity between the dentate gyrus (DG) and field CA3 in euploid mice (A), euploid mice treated with fluoxetine (B), Ts65Dn mice (C) and Ts65Dn mice treated with fluoxetine (D). Ts65Dn mice have a reduced number of granule cells (GC), mossy fibers (MF), glutamatergic terminals (GT) in the stratum lucidum and spines (SP) on the thorny excrescences (C). Treatment with fluoxetine rescues all these defects and restores the input from the DG to CA3 (D). In euploid mice treatment increases the glutamatergic terminals and spine density, with no effect on the number of granule cells and mossy fibers (B). The direction of the arrows indicates the direction of the defect and the effect of the therapy in comparison with untreated euploid mice. The double-headed arrows indicate no effect.

The fluoxetine-induced rescue of the total number of granule neurons in Ts65Dn mice [12] is an essential but not sufficient condition for the functional recovery of the trisynaptic circuit. It is equally important that the surplus of granule cells establishes appropriate synaptic contacts with CA3, the second element of the trisynaptic circuit. We found here that in trisomic mice treated with fluoxetine there was the recovery of the input from the DG to CA3. This recovery includes restoration of i) the number of granule cells and, hence, of mossy fibers; ii) the density of glutamatergic terminals in the stratum lucidum; and, iii) the density of spines forming the thorny excrescences (Fig. 10). In addition, electrophysiological evidence showed that the increased excitatory connectivity was functionally effective and that the inhibitory input to CA3 was also restored.

Neuron generation and dendritic maturation with consequent alterations in connectivity are heavily compromised in DS. Thus, therapies to improve brain development should be aimed at restoring all these processes. Treatment with fluoxetine in adult Ts65Dn mice has been shown to restore neurogenesis in the DG [21]. No studies have examined the effects of fluoxetine during adulthood on dendritic architecture and connectivity. It should be noted that treatments in adulthood are likely to moderately impact on brain cellularity and wiring that mainly occur in the very early phases of brain development. We previously found that neonatal treatment with fluoxetine fully restores hippocampal neurogenesis [12], dendritic architecture and spine density of trisomic granule cells [16], indicating that the same treatment is able to restore not only the number of granule neurons but also their “quality” in terms of correct maturation. Moreover, the rescue of dendritic development was accompanied by the rescue of connectivity to the DG [16]. Current findings show that fluoxetine restores connectivity between the DG and field CA3 of Ts65Dn mice. Taken together, these data suggest that early treatment with fluoxetine restores the organization of the hippocampal circuits.

The widespread beneficial effects of fluoxetine on the hippocampal formation and the finding that postnatal pharmacotherapy with fluoxetine restores cognitive performance [12] suggest that early treatment with fluoxetine can be a suitable therapy, possibly usable in humans, to restore the physiology of the hippocampal networks and, hence, memory functions.
